# Supplementary material for: Injury-induced intestinal stem cell renewal requires capillary morphogenesis gene 2
Source: EMBO Mol Med. 2025 Aug 22;17(10):2612–31. doi: 10.1038/s44321-025-00295-3 (PMC12514029; doi:10.1038/s44321-025-00295-3)
Supplement: Supplementary file 2 — Appendix [file 44321_2025_295_MOESM2_ESM.pdf]

APPENDIX

Injury-Induced Intestinal Stem Cell Renewal Requires Capillary Morphogenesis Gene 2

Lucie Bracq[1*], Audrey Chuat[1], Béatrice Kunz[1], Olivier Burri[2], Romain Guiet[2], Julien Duc[3], Nathalie Brandenberg[1] and F. Gisou van der Goot[1*]

[1] Global Health Institute, School of Life Sciences, [2] BioImaging and Optics Core Facility, School of Life Science, EPFL, Lausanne Switzerland. 3Nexco Analytics, EPFL Innovation Park, Lausanne, Switzerland.

Appendix Figure S1 .... Page 2
Appendix Figure S2 .... Page 3
Appendix Figure S3 .... Page 4-5
Appendix Methods .... Page 6-9
  Animal's characteristics .... Page 6-7
  Animal care and monitoring .... Page 8
  Microscope technical specifications: .... Page 9

Appendix Figure S1

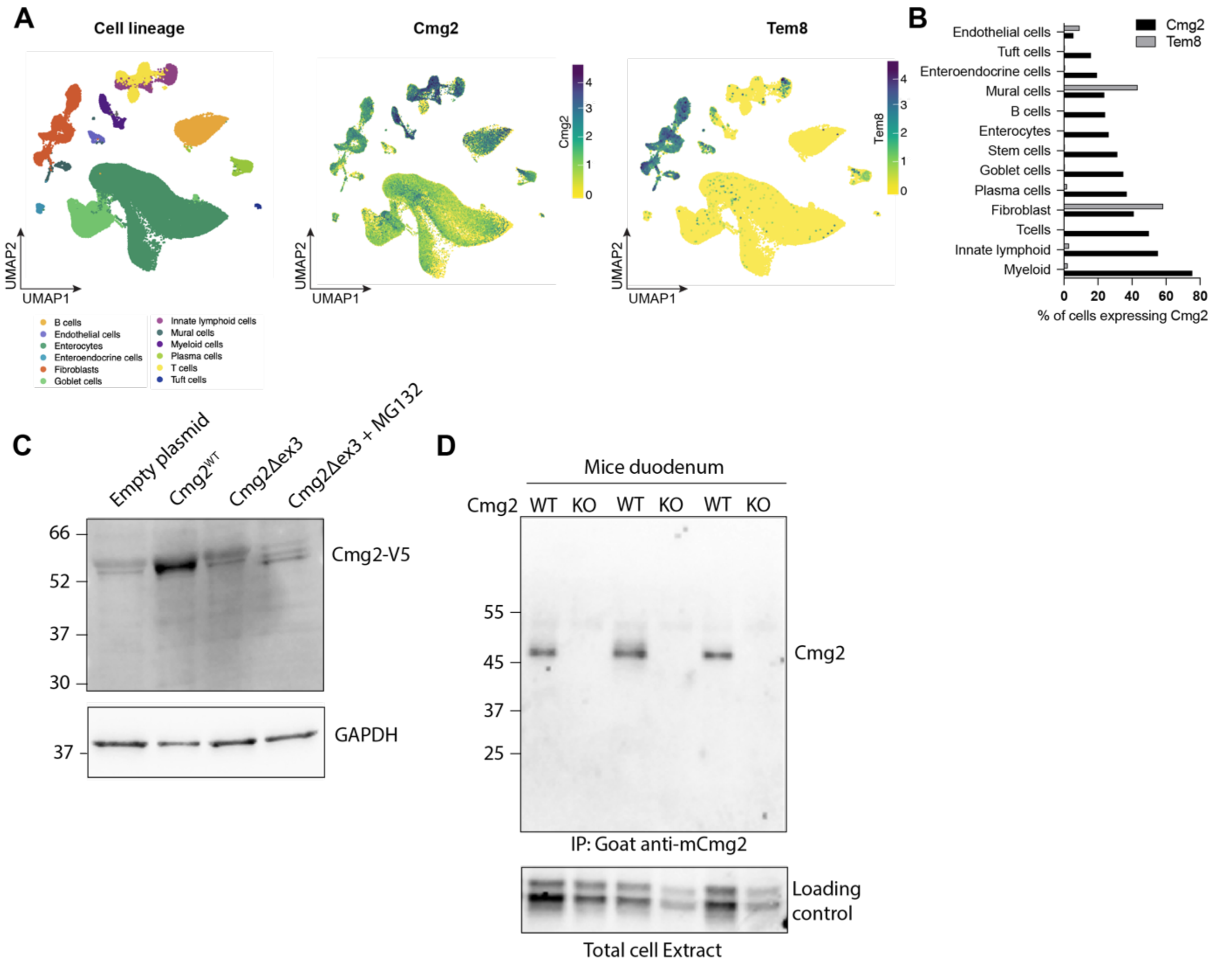

Appendix Figure S1: Cmg2 and Tem8 expression pattern in the gut. (A) UMAP of scRNAseq replotted from raw data published in Mayassi et al., 2024 (GEO accession GSE245316). (B) Percentage of cells expressing Cmg2 or Tem8 from scRNAseq (GEO accession GSE245316). (C) Western blot analysis of CMG2-V5 expressed in RPE-1 cells transfected with either an empty plasmid, wild-type CMG2 (Cmg2WT), or a CMG2 construct lacking exon 3 (Cmg2Δex3), in the absence or presence of the proteasome inhibitor MG132. CMG2-V5 was detected using an anti-V5 antibody. GAPDH was used as a loading control. (D) CMG2 expression in duodenal tissue from wild-type (WT) and Cmg2 knockout (KO) mice. CMG2 was immunoprecipitated using a goat anti-mouse CMG2 antibody prior immunoblotting against Cmg2. Lower panel: total cell extract (input) shown as a loading control.

Appendix Figure S2

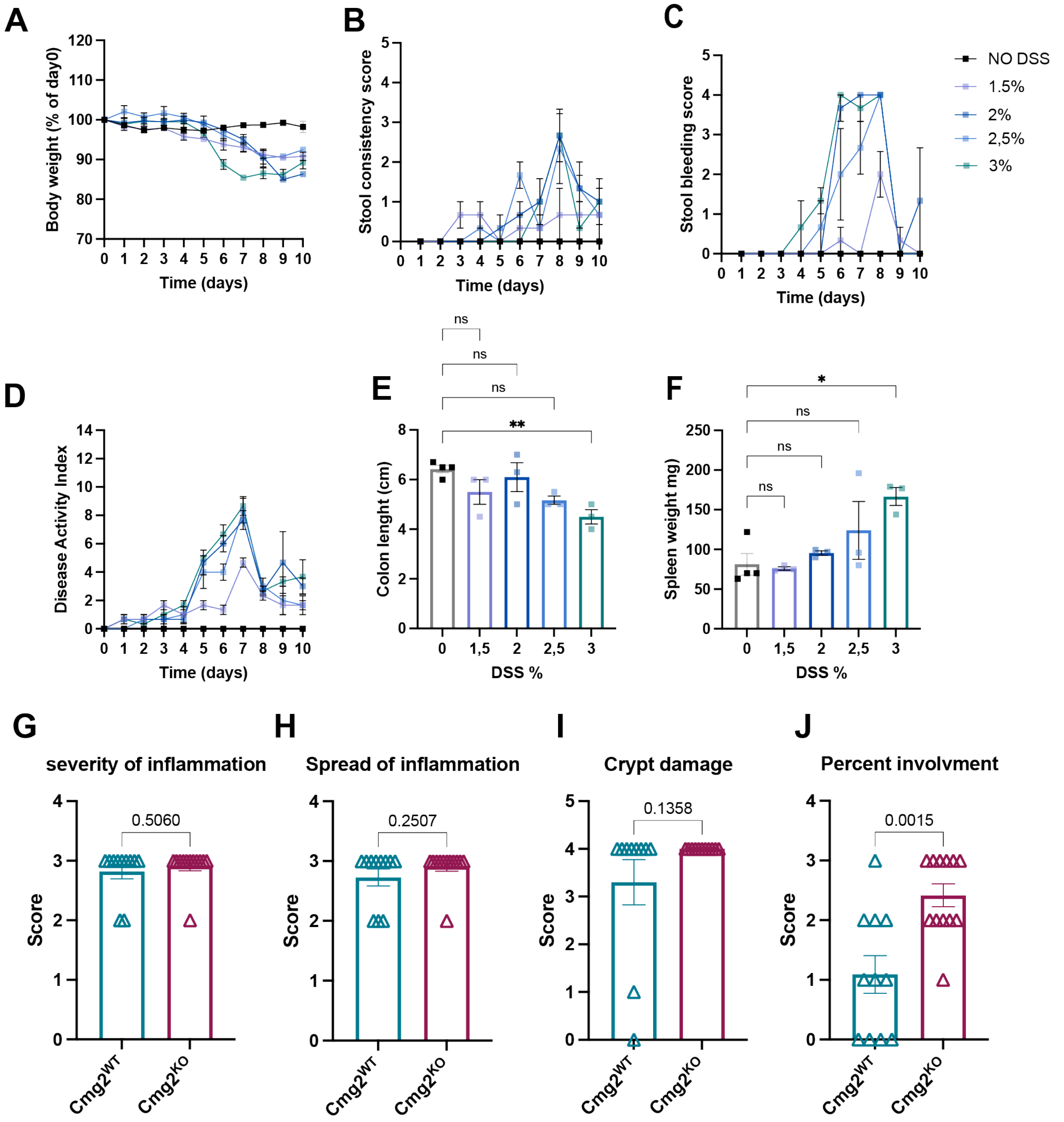

Appendix Figure S2: Pilot experiment and histological score. (A-F) Pilot experiment in which 8-week-old *Cmg2*^{WT} males were given 1.5 to 3% Dextran-Sulfate-Sodium (DSS) in their drinking water for 7 days then switched to regular drinking water for 3 days to recover and monitored daily during the 10day experiment for (A) Body weight loss, (B) the aspect of the feces and (C) presence of occult blood used to determine (D) Disease activity index (DAI) scoring. On day 10, mice were euthanasied and colon and spleen collected and (E) measured or (F) weighted respectively. (G-I) Detailed histological score shown in Fig3d.

Appendix Figure S3

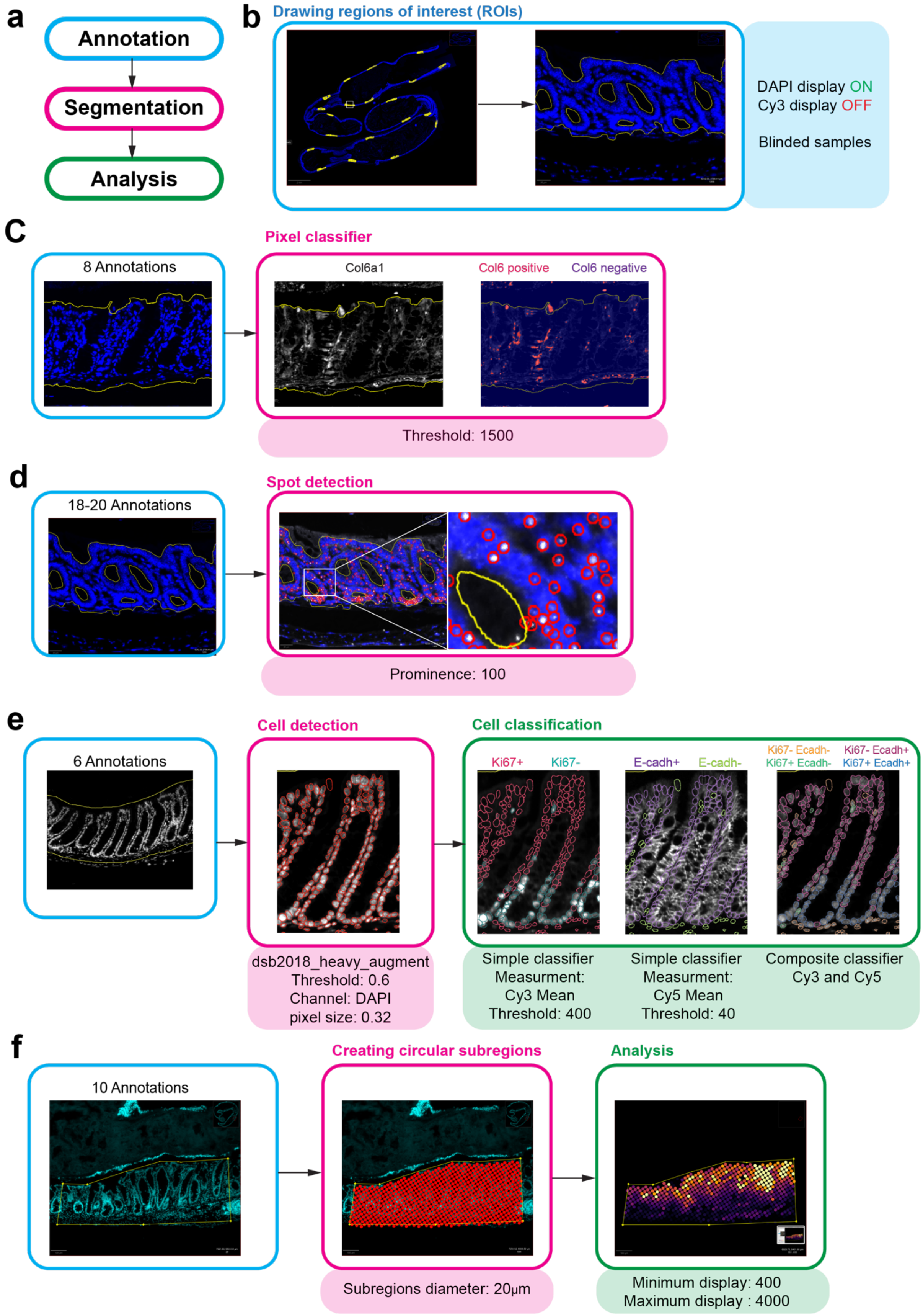

Appendix Figure S3: Microscopy workflow: (a) Microscopy workflow follow three different steps including annotation, segmentation and analysis. **(b)** Annotation of region of interest is performed on blinded samples (blind genotype and mice id). During this step, channels of interest are turned off and annotations are drawn manually using the DAPI channels. 6 to 20 annotations per sample are drawn along the whole tissue section. **(c)** To quantify the percentage of Col6a1+ tissue area, annotations are drawn as described and Qupath pixel classifier is performed. Threshold is set by visual inspection and applied to all samples. **(d)** For spot detection, annotations are drawn and script RNAscope_Count has been used to quantify the number of spots per annotation area. Prominence is set by visual inspection and apply to all samples. **(e)** To quantify the percentage of E-Cadherin+ and Ki67+ cells, annotations were drawn and cells detected using DAPI channel and Qupath STARDIST. Pixel classifiers were then used to classify cells accordingly to E-cadherin and Ki67 staining. **(f)** To analyze Ly6a in colon section, annotations were divided in multiple circular subregions of 20um diameter. The mean fluorescence intensity of Ly6a in each subregion was then quantify and shown using Qupath display.

Supplementary information

Animal's characteristics

Animals involve in DSS experiments:

				BW at start				Age at start (weeks)			
		n	sex	mean	SD	SEM	p-value	mean	SD	SEM	p-value
Group A	WT Ctrl	8	100% male	27.6	1.554	0.5495		8.429	0.187	0.06613	
Group B	KO Ctrl	8	100% male	27.24	1.735	0.6135	0.9845	8.536	0.2504	0.08852	0.8928
Group C	WT DSS 7	4	100% male	27.8	1.169	0.5845	0.9997	8.857	0.4041	0.202	0.0527
Group D	KO DSS 7	5	100% male	26.08	1.487	0.6651	0.289	8.829	0.5477	0.2449	0.0519
Group E	WT DSS 7+3	15	100% male	27.55	1.355	0.3498	>0.9999	8.638	0.1608	0.04151	0.2831
Group F	KO DSS 7+3	15	100% male	26.47	1.643	0.4243	0.3194	8.619	0.2513	0.06489	0.3687

Table Analyzed	All mice used - BW at start				
Data sets analyzed	A-F				
**ANOVA summary**					
F	1.568				
P value	0.1867				
P value summary	ns				
Significant diff. among means (P < 0.05)?	No				
R squared	0.1379				
**Brown-Forsythe test**					
F (DFn, DFd)	0.2490 (5, 49)				
P value	0.9383				
P value summary	ns				
Are SDs significantly different (P < 0.05)?	No				
**Bartlett's test**					
Bartlett's statistic (corrected)	1.079				
P value	0.956				
P value summary	ns				
Are SDs significantly different (P < 0.05)?	No				
**ANOVA table**	**SS**	**DF**	**MS**	**F (DFn, DFd)**	**P value**
Treatment (between columns)	18.31	5	3.662	F (5, 49) = 1.568	P=0.1867
Residual (within columns)	114.4	49	2.336		
Total	132.8	54			
**Data summary**					
Number of treatments (columns)	6				
Number of values (total)	55				

Number of families	1							
Number of comparisons per family	5							
Alpha	0.05							
**Dunnett's multiple comparisons test**	**Mean Diff.**	**95.00% CI of diff.**	**Below thresh**	**Summary**	**Adjusted P Value**		**A-?**	
WT Ctrl vs. KO Ctrl	0.3687	-1.616 to 2.353	No	ns	0.9845		B	KO Ctrl
WT Ctrl vs. WT DSS 7	-0.1963	-2.627 to 2.235	No	ns	0.9997		C	WT DSS 7
WT Ctrl vs. KO DSS 7	1.524	-0.7392 to 3.787	No	ns	0.289		D	KO DSS 7
WT Ctrl vs. WT DSS 7+3	0.05042	-1.687 to 1.788	No	ns	>0.9999		E	WT DSS 7+3
WT Ctrl vs. KO DSS 7+3	1.13	-0.6074 to 2.868	No	ns	0.3194		F	KO DSS 7+3
**Test details**	**Mean 1**	**Mean 2**	**Mean Diff.**	**SE of diff.**	**n1**	**n2**	**q**	**DF**
WT Ctrl vs. KO Ctrl	27.6	27.24	0.3687	0.7641	8	8	0.4826	49
WT Ctrl vs. WT DSS 7	27.6	27.8	-0.1963	0.9359	8	4	0.2097	49
WT Ctrl vs. KO DSS 7	27.6	26.08	1.524	0.8712	8	5	1.749	49
WT Ctrl vs. WT DSS 7+3	27.6	27.55	0.05042	0.6691	8	15	0.07535	49
WT Ctrl vs. KO DSS 7+3	27.6	26.47	1.13	0.6691	8	15	1.69	49

Other mice used in this study:

Mice used for BW follow-up		n	sex
Group A	WT	9	100% male
Group B	KO	8	100% male

Additional Ctrl Mice used		n	sex	Age at start (weeks)			
				**mean**	**SD**	**SEM**	**p-value**
Group A	WT	12	100% male	10.37	0.5221	0.1507	
Group B	KO	12	100% male	10.19	0.2608	0.07529	0.3

Table Analyzed	
Column B	KO
vs.	vs.
Column A	WT
**Unpaired t test**	
P value	0.3
P value summary	ns
Significantly different (P < 0.05)?	No
One- or two-tailed P value?	Two-tailed
t, df	t=1.060, df=22
**How big is the difference?**	
Mean of column A	10.37
Mean of column B	10.19
Difference between means (B - A) ± SEM	-0.1786 ± 0.1685
95% confidence interval	-0.5280 to 0.1708
R squared (eta squared)	0.04859
**F test to compare variances**	
F, DFn, Dfd	4.007, 11, 11
P value	0.03
P value summary	*
Significantly different (P < 0.05)?	Yes
**Data analyzed**	
Sample size, column A	12
Sample size, column B	12

Animal care and monitoring

During DSS experiment, animals care and welfare was monitored accordingly to the following scoresheet validated by the approved by the Veterinary Authorities of the Canton Vaud and according to the Swiss Law (License VD 3497, EPFL).

					on handling	
**1. Parameters:**	**haircoat**	**eyes and nose**	**breath**	**posture**	**activity**	**body weight loss over 10 days experiment**
	0 = normal, well-groomed	0 = normal	0 = normal, deep breathing	0 = normal posture	0 = normal move around the cage	0 = no weight loss
**2. Score points (description)** gradation of the severity of the expected symptoms	1 = fur ruffling	1 = eyes closed or squinted, no discharge	1 = shallow	2 = Sporadic hunchback posture	1 = decrease activity locomotion after slight	1 = 1–10% weight loss
	2 = fur ruffling, urine-stained hair coat, ungroomed	2 = chromodachryorrhea	2 = labored breathing, hyperventilation	3 = frequent or permanent hunched position	2 = inactive, lethargy, locomotion after moderate stimulation	2 = 10–15% weight loss
					3 = Ataxia, immobile, no locomotion after moderate stimulation	3 = 15–25% weight loss
						4 = > 25% weight loss

3. Endpoints:

3.1: critcal score:euthanize if any score 3, excepted score 4 for body weight loss

3.2: total score euthanize if sum of general score points > 6

3.3: time course: max. duration of a defined score: euthanize if score of 2 or more during the second control of the day for labored breathing or hunched position (exept weight)

4. Checking (Frequency):

Every day

Twice a day if parameter are elevated = 2 (except 3 for weight)

Microscope technical specifications:

Stand: Upright Olympus motorized custom microscope.
XY-Stage: Motorized
Illumination: X-Cite NOVEM XT-920
Software: Slideview VS 200

Detectors

Cameras	Color UI 3280 CP	Black/White VS20-304M
Chip Technology	CMOS Color	CMOS Grayscale
Sensor Format	2456 x 2054	4104 x 3006
Chip size	8.47mm x 7.08mm	14.15mm x 10.37mm
Pixel size	3.45 um x 3.45 um	3.45 um x 3.45 um (used at 2x binning)
Dynamic Range	8-bit	12-bit
C-mount	0.63x	1.0x

Objectives

Objective	Mag/NA	Medium	Contrast	WD (mm)
PLAPON	2x/0.08	air	Pol	6.20
UPLXAPO	4x/0.16	air	Pol	13.00
UPLXAPO	10x/0.40	air	Pol	3.10
UPLXAPO	20x/0.80	air	Pol	0.60
UPLXAPO	40x/0.95	air	Pol	0.18
UPLXAPO	40x/1.40	oil	Pol	0.13

Filtercubes

The links in the table below will send you to an FPbase configuration page.

Penta Band Filter: Custom F66-987_OEFF (AHF-LED-DFC3C5C7-5-SBM)

Name	Excitation	Dichroic	Emission	Nº
DAPI	BP 378/52	Pentaband BP	BP 432/36	FF01-378/52-25 FF01-432/36-25
FITC	BP 474/27	Pentaband BP	HC 515/30	FF01-474/27-25 FF01-515/30-25
Cy3	BP 554/23	Pentaband BP	HC 595/31	FF01-554/23-25 FF01-595-31-25
Cy5	BP 635/18	Pentaband BP	BP 685/40	FF01-635/18-25 FF02-685/40-25
Cy7	BP 735/28	Pentaband BP	BP 809/81	FF01-735/28-25 FF02-809/81-25

Full spectrum for all filters
