# Supplementary material for: Injury-induced intestinal stem cell renewal requires capillary morphogenesis gene 2
Source: EMBO Mol Med. 2025 Aug 22;17(10):2612–31. doi: 10.1038/s44321-025-00295-3 (PMC12514029; doi:10.1038/s44321-025-00295-3)
Supplement: Supplementary file 4 — Expanded View Figures [file 44321_2025_295_MOESM4_ESM.pdf]

Expanded View Figures

Figure EV1. Cmg2 KO mice have normal guts under basal conditions.

(**A**) Body weight of Cmg2^{WT} and Cmg2^{KO} over a 30 weeks period. Results are mean ± SEM. Each dot represents the mean of at least $n = 8$ mice per genotype. (**B**) Colon tissues under basal conditions, from 8-weeks-old *Cmg2*^{WT} and *Cmg2*^{KO} mice were stained for Hematoxylin/Eosin, Sirius Red and Alcian Blue. Representative images of at least $n = 8$ mice per genotype. Scale bar, 20 µm. (**C**) Number of crypts per 800 µm of tissue screened, (**D**) crypt lengths were quantified and showed as superplot. Results are mean ± SEM. Each dot represents a single measurement and each triangle represent the mean per mouse. At least $n = 5$ mice per genotype were quantified. *P* values obtained by unpaired two-tailed *t* test. (**E**) Colon lysates were analyzed by SDS–PAGE using 4–12% Bis-Tris gradient gels under reducing condition and western blotted against all the collagen VI. Migration of the molecular weight markers (in kDa) are indicated on the left. (**F**) CollagenVI/loading control ratio were quantified and normalized to the mean of Cmg2^{WT}. Results are mean ± SEM, and $n = 12$ mice per genotype were quantified. *P* values obtained by unpaired two-tailed *t* test. (**G**) Colon tissues were immunostained with anti-collagen VI and DAPI. Representative image of $n = 6$ mice per genotype. Scale bar, 20 µm. (**H**) % of Collagen VI area per tissue section was quantified on $n = 6$ mice per genotype. Results are mean ± SEM. Each dot represents a single measurement and each triangle represent the mean per mouse. *P* values obtained by unpaired two-tailed *t* test. q PCR analysis of colonic tissues for (**I**) Col6a1, and (**J**) ISC markers Lgr5, Ascl2, Axin2 and Cyclin D1 genes were performed on $n = 8$ mice per genotype. *P* values obtained by unpaired two-tailed *t* test in (**I**) and by two-way ANOVA Šídák's multiple comparisons test in (**J**). (**K**) Colonic tissues were stained for RNAscope in situ hybridization against Lgr5 and (**L**) number of Lgr5 mRNA spots per µm^2 were quantified. (**M**) Colonic sections were stained anti-ki67, anti-E-cadherin and DAPI. (**N**) % of E-cadherin+ epithelial cells, and (**O**) % of E-cadherin + /Ki67+ epithelial cells, were quantified on $n = 8$ mice per genotype. *P* values obtained by unpaired two-tailed *t* test. (**P**) qPCR analysis of colonic tissues for inflammatory cytokines genes was performed on $n = 8$ mice per genotype. *P* values obtained by two-way ANOVA Šídák's multiple comparisons test.

 © The Author(s)

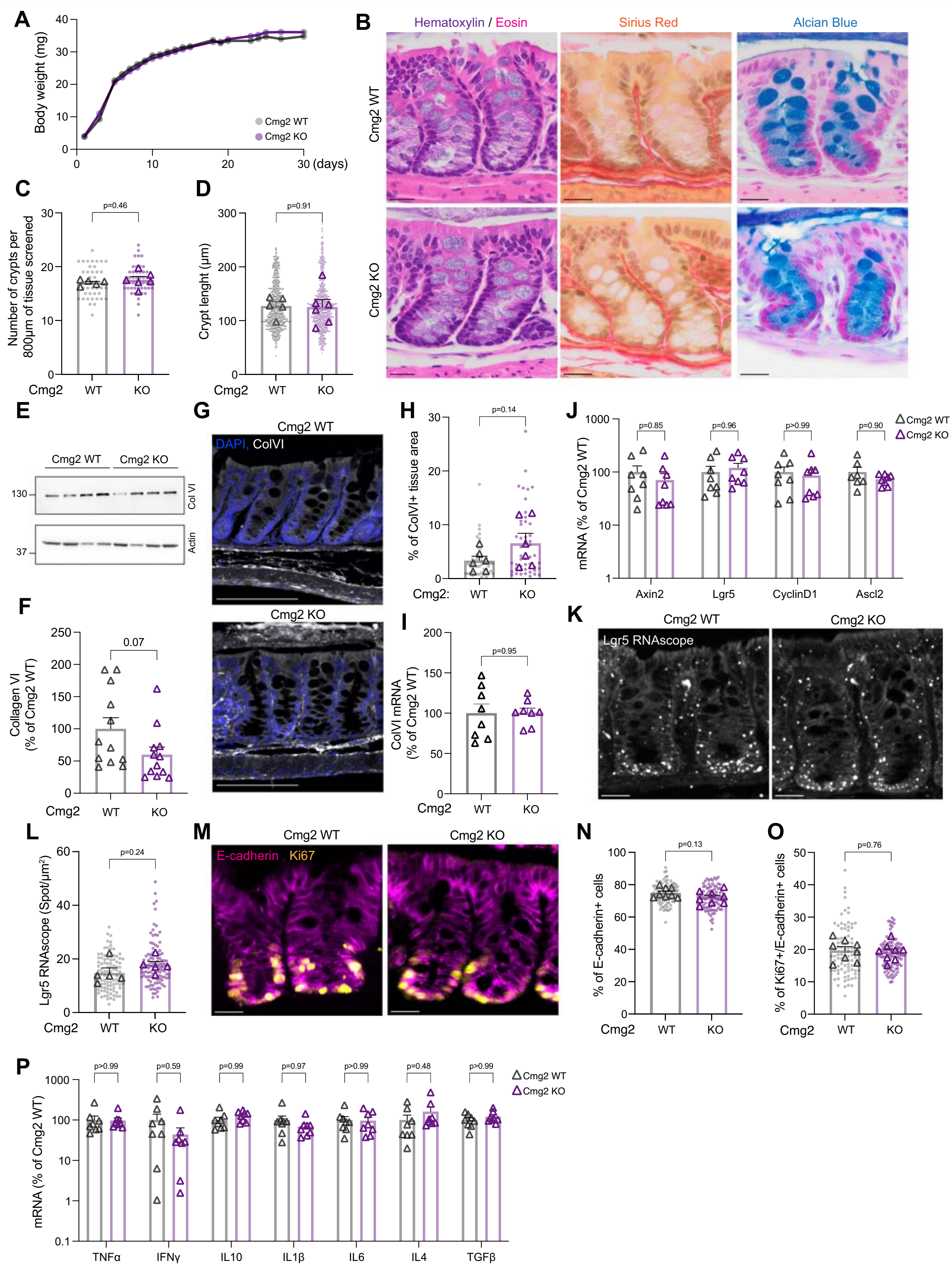
A
Body weight (mg)
Cmg2 WT
Cmg2 KO
30 (days)
B
Hematoxylin / Eosin
Sirius Red
Alcian Blue
Cmg2 WT
Cmg2 KO
C
Number of crypts per 800µm of tissue screened
p=0.46
Cmg2 WT KO
D
Crypt lenght (µm)
p=0.91
Cmg2 WT KO
E
Cmg2 WT
Cmg2 KO
130
37
Col VI
Actin
F
Collagen VI (% of Cmg2 WT)
0.07
Cmg2 WT KO
G
Cmg2 WT
DAPI, ColVI
Cmg2 KO
H
% of ColVI+ tissue area
p=0.14
Cmg2: WT KO
I
ColVI mRNA (% of Cmg2 WT)
p=0.95
Cmg2 WT KO
J
mRNA (% of Cmg2 WT)
p=0.85
p=0.96
p>0.99
p=0.90
Axin2
Lgr5
CyclinD1
Ascl2
K
Cmg2 WT
Cmg2 KO
Lgr5 RNAscope
L
Lgr5 RNAscope (Spot/µm²)
p=0.24
Cmg2 WT KO
M
Cmg2 WT
Cmg2 KO
E-cadherin, Ki67
N
% of E-cadherin+ cells
p=0.13
Cmg2 WT KO
O
% of Ki67+/E-cadherin+ cells
p=0.76
Cmg2 WT KO
P
mRNA (% of Cmg2 WT)
p>0.99
p=0.59
p=0.99
p=0.97
p>0.99
p=0.48
p>0.99
TNFα
IFNγ
IL10
IL1β
IL6
IL4
TGFβ

© The Author(s)

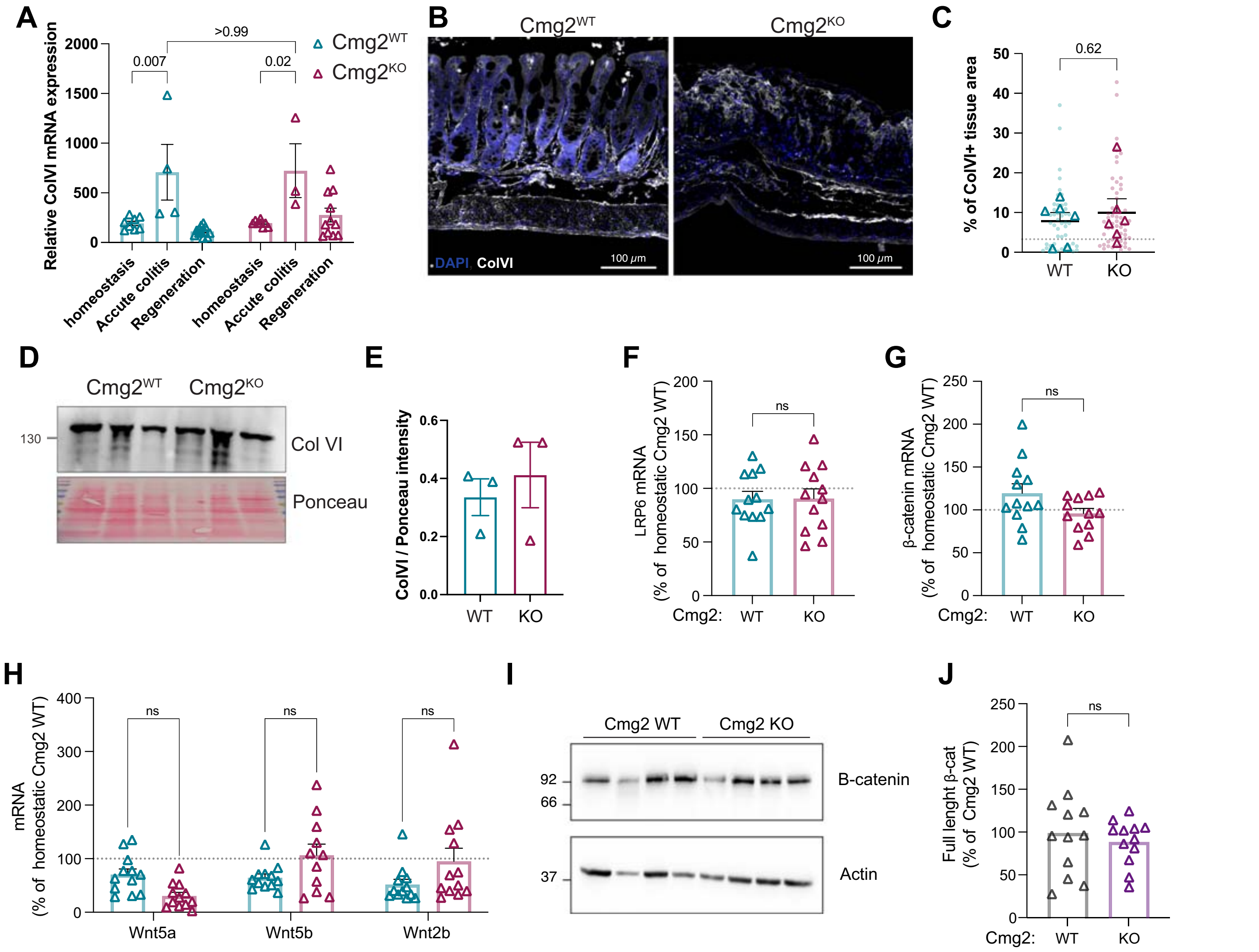

Figure EV2. Collagen and Wnt players in Cmg2^{KO} mice.

(**A**) qPCR analysis of colonic tissues from *Cmg2*^{WT} and *Cmg2*^{KO} during homeostasis, after 7days of DSS or 3 days after DSS withdrawal for Col6a1. Results are mean ± SEM. Each symbol represents the mean per mouse. At least $n = 4$ mice per genotype were quantified. *P* values obtained by two-way ANOVA Šídák's multiple comparisons test. (**B**) Colon tissues from Cmg2^{WT} and Cmg2^{KO} mice 3 days after DSS withdrawal (day 10) were immunostained with anti-collagen VI and DAPI. Representative image of $n = 6$ mice per genotype. Scale bar, 100 µm. (**C**) % of Collagen VI area per tissue section was quantified on $n = 6$ mice per genotype. Results are mean ± SEM. Each dot represents a single measurement and each triangle represent the mean per mouse. *P* values obtained by unpaired two-tailed *t* test. (**D**) colonic lysates from Cmg2^{WT} and Cmg2^{KO} mice 3 days after DSS withdrawal (day 10) were analyzed by SDS-PAGE using 4–12% Bis-Tris gradient gels under reducing condition and western blotted against all the collagen VI. Migration of the molecular weight markers (in kDa) are indicated on the left. (**E**) CollagenVI/Ponceau ratio were quantified. Results are mean ± SEM, and $n = 3$ mice per genotype were quantified. (**F–H**) qPCR analysis of colonic tissues from Cmg2^{WT} and Cmg2^{KO} mice 3 days after DSS withdrawal (day 10) for (**F**) LRP6, (**G**) β-catenin and (**H**) Wnt ligand, Results are mean ± SEM. Each symbol represents the mean per mouse. The dotted line represents the mean of homeostatic Cmg2^{WT} mice. $n = 12$ mice per genotype was quantified. *P* values obtained by two-tailed unpaired *t* test in (**F**, **G**) or by two-way ANOVA Šídák's multiple comparisons test in (**H**). (**I**) Colon lysates were western blotted against β-catenin. (**J**) β-catenin/loading control ratio were quantified and normalized to the mean of Cmg2^{WT}. Results are mean ± SEM, and $n = 12$ mice per genotype were quantified. Each triangle represents the mean per mouse. *P* values obtained by unpaired two-tailed *t* test.

 © The Author(s)

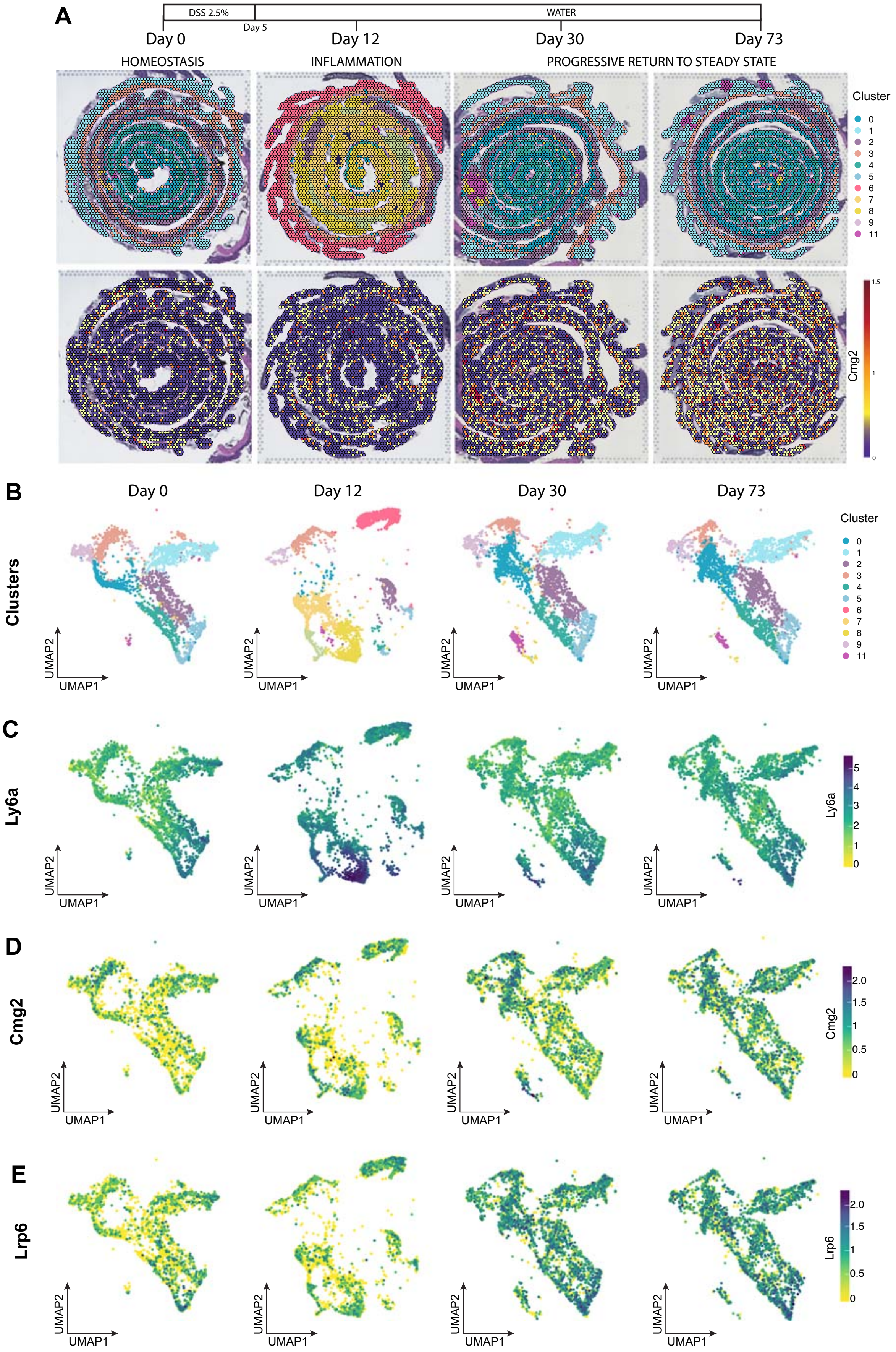
A
DSS 2.5%
WATER
Day 5
Day 0
Day 12
Day 30
Day 73
HOMEOSTASIS
INFLAMMATION
PROGRESSIVE RETURN TO STEADY STATE
Cluster
0 1 2 3 4 5 6 7 8 9 11
Cmg2
B
Day 0
Day 12
Day 30
Day 73
Clusters
UMAP1
UMAP2
C
Ly6a
D
Cmg2
E
Lrp6

Figure EV3. Spatial transcriptomics of DSS-treated mice throughout the post-treatment recovery course for up to 73 days.

(**A**) Clusters of VISIUM spots (upper panel) or Cmg2 expression (lower panel) coupled with tissue H&E staining, replotted from raw data published in Mayassi et al, 2024 (Data ref: Mayassi et al, 2024a). (**B**) UMAP clustering of VISIUM data. (**C–E**) Ly6a (**C**), Cmg2 (**D**) and Lrp6 (**E**) expression on intestinal tissue from VISIUM data.
